# Optimal Steps for designing and implementing the extracurriculars through the integrative medical approach

**DOI:** 10.1016/j.heliyon.2023.e13755

**Published:** 2023-02-15

**Authors:** Ihab Shafek Atta, Ali Hendi Alghamdi, Rajab A. Alzaharni

**Affiliations:** a Pathology Department, Faculty of Medicine, Al-Azhar University, Assuit, Egypt; b Pathology Department, Faculty of Medicine, Al-Baha University, Saudi Arabia; c Ophthalmology Division, Surgery Department, Faculty of Medicine, Al-Baha University, Saudi Arabia; d Otolaryngology Division, Surgery Department, Faculty of Medicine, Al-Baha University, Albaha, Saudi Arabia

**Keywords:** Academic performance, Extracurriculars, Educational evaluation, Integrated curriculum, Kern’s steps

## Abstract

Extracurriculars are those that fall outside the scope of the academic curriculum. The purpose of the work is to outline the steps of planning extracurriculars, practice these steps in the medical program, and evaluate these steps.

**Methods:**

Using Kern’s steps with some modifications, we did some extracurricular reforms. Assessment of the current situation/needs and identification of gaps have been occurred by a questionnaire that revealed low students' satisfaction (36.1%) about the current extracurriculars with points of weakness that have been addressed in the improvement plan. A list of extracurriculars was prepared and aligned with modules and learning outcomes. Allocation of resources and implementation of these extracurriculars were performed. The evaluation was done through a questionnaire that was fulfilled by 404 students.

**Results:**

Students' satisfaction was 66.8% in the second questionnaire compared to 36% in the initial questionnaire with a significant association. Further analysis of the respondents who revealed satisfaction showed that 95 out of 140 (67.8%) were high-grade achievers, 88 out of 134 (65.7%) for moderate, and 87 out of 130 (66.9%) in low-grade achievers. A comparison of the student’s satisfaction in the three phases revealed a significant p-value (0.004), but no significance in students' satisfaction within phases of the program between males and females.

**Conclusion:**

Well-structured extracurriculars might contribute to the achievement of the mission, vision, and goals of the program. Extracurricular activities might be flexible and undergo periodic changes related to the nature of the curriculum. Following the cycle of developing extracurricular activities in designing, implementing, monitoring, evaluating, and reporting, the extracurricular activities will be more efficient in enhancing the learning climate and making the learning process more enjoyable, especially in a solid medical integrated curriculum.

## Highlights

• Foundation of an extracurricular committee is necessary for monitoring the whole cycle of extracurricular activities.
• Extracurriculars should be aligned with the objectives of the modules and courses of the curriculum.
• Good extracurricular implantation with appropriate timing will enhance the learning climate and students’ performance.

## 1 Introduction

Extracurricular activities are those that fall outside the scope of the academic curriculum of university education. They comprised participation in community medical volunteer services, nonmedical-related community services, club events, government-related activities, research plans, and competitive academic or nonacademic teams that have competitions that occur at all levels, from local to national and even international [1]. However, in medical schools, the extracurricular activity mostly valued is community-related medical services [2].

As such, extracurricular activities may constitute a haven where students aim to utilize, refine, and develop their interpersonal skills, communication skills, and practical skills [3,4]. Furthermore, extracurricular activities can reduce anxiety, stress, and burnout and their effects on mental and physical health [5].

Furthermore, students who participated in extracurricular activities showed an elevated level of competence, confidence, accomplishment, self-esteem, and psychosocial development, which is more marked in the educational state, cultural participation, life management, and career planning [6]. Another study revealed that those who have their place in clubs/organizations are expected to acquire more interpersonal skills, and those with community-related activities are more socializers than others [4]. Students' participation in extracurriculars has been linked positively with academic performance [[7], [8], [9]]. However, students may desist from participating to study more, save time and attain high grades [4,10,11].

Da Silveira et al. [12] reported that students who shared in the extracurriculars revealed high levels in the cognitive and affective domain, while students who did not participate remained in the lowest level. Other studies revealed that the engagement of students in extracurriculars has been found to increase both the attendance of students and their grade point average [[13], [14], [15], [16]]. However, few studies revealed no improvement in the student’s achievement in students sharing these activities [[17], [18], [19], [20]].

The diversity of extracurricular activities and the benefits obtained from them have been researched considering the traditional curricula, but they have not been professionally researched within the framework of the integrative medical approach. Since the College of Medicine, Al-Baha University, pursues the integrative medical approach in all stages of the program, it was necessary to study the feasibility of these extracurricular activities and reformulate and organize them to contribute to achieving program outcomes effectively.

As it is known previously through the published literature [[3], [4], [5], [6]], the extent of the benefits of these activities and their importance for the student, the program, and the educational environment, and in order for these benefits to be achieved, they must be ideally employed in terms of how they are chosen, their purpose, methods of implementation, and the selection of the appropriate time and duration in proportion to achieving the goal and not being affected by the educational process.

The original goal in developing extracurricular activities is to serve the curriculum as well as help in achieving the educational outcomes of the program in addition to entertaining students and raising their moral and psychological status as well as breaking barriers and increasing communication between them and faculty members and program management [11].

However, in many cases, extracurricular activities are placed within the framework of entertainment rather than those that serve the integrative curriculum. It has been noted that placing these activities in an incorrect framework may negatively affect the achievement of the educational outcomes of the program and the students' performance.

Most of the studies conducted in this field were either focused more on the importance of choosing these activities in the pre-university stages in order to enroll in medical colleges [[21], [22], [23], [24]], or they were conducted in other traditional programs and were never conducted in an integrative medical approach. Therefore, this research is considered original and new and has a precedent in this field.

For this study to be comprehensive for all students of all levels, it was necessary to know their academic grade point average (GPA) and make a comparison between their level of satisfaction and their academic average so that we can prove that the results obtained, whether positive or negative, do not only stem from a category, but include all students at their different academic levels.

Are the extracurricular activities in their current condition suitable for the integrative medical approach? Are they capable of achieving the desired goals for both the program and the students? Are there any obstacles that prevent their optimal implementation? All these questions must be answered within the framework of the integrative approach.

Therefore, the main purpose of this research includes several goals, the first of which is to investigate the extent of students' satisfaction with these extracurricular activities in the current situation. Secondly, developing an appropriate plan to address these causes within the framework of the improvement plan, by taking advantage of the steps that Kern followed [25]. Thirdly, achieving these steps through an applied procedure for those extracurricular activities in all stages of the program. Fourth, re-evaluation of students' satisfaction with these extracurricular activities after implementing the improvement plan. Fifth. Comparing the level of student satisfaction before and after the implementation of the improvement plan.

## 2 Methods

The study was conducted in faculty of medicine, Al-Baha university under ethical approval no. 62/2020 during two academic years (2020–2021) and (2021–2022).

A. Study design

This study consisted of four phases

1) An initial cross-sectional quantitative study by setting an initial survey through Likert scale questionnaire that was conducted electronically to students to investigate the extent of their satisfactions about the current extracurricular activity. The dependent variables include students' satisfaction, students' performance and independent variables were goals, consistency with modules/courses, timing, duration, facilities, allocation, transport, and availability of other logistic materials.
2) Designation, and implementation of the improvement plan through modification of kern' steps [25] for planning the extracurricular activities.
3) Re-evaluation of students' satisfaction with these extracurricular activities after implementing the improvement plan using second questionnaire with Likert scale.
4) Comparing the level of student satisfaction before and after the implementation of the improvement plan.

B. Study sample:

The number of respondents in the initial questionnaire was 184, 116 male (63%) and 68 females (37%) (Table 1), while in the re-evaluation phase the number of respondents was 404 students representing all academic years for both male and female sections.

C. Data collection and statistically analysis

**Table 1:** Distribution of the degree of satisfaction concerning gender and program phase in the phase of assessment of current situation.

Phase of the program	Gender	Total	Strongly satisfied		Satisfied		Dissatisfied		Strongly Dissatisfied		P value (*t*-test)
			No.	%	No.	%	No.	%	No.	%	
	Male	116	26	22.4	16	13.7	42	36.2	32	27.6	0.2132
	Female	68	14	20.6	15	22	20	29.4	19	22.1	
I	Male	33	8	24.2	4	12.1	13	39.4	8	24.2	
	Female	17	3	17.6	4	23.5	4	23.5	6	35.3	
II	Male	45	8	17.8	7	15.6	18	40	12	26.7	
	Female	28	6	21.4	7	25	9	32.1	6	21.4	
III	Male	38	10	26.3	5	13.2	11	28.9	12	31.6	
	Female	23	5	21.7	4	17.4	7	30.4	7	30.4	

The questionnaire was designed as Likert scale containing four categories started from strongly satisfied to strongly dissatisfied and scored from 4 to 1. Data from the questionnaires were tested for reliability and placed into an Excel sheet before being analyzed statistically with SPSS version 25. Student *t*-test and ANOVA were run for inferential statistics.

## 3 Mechanism of designing of modified Kern' steps used in this study

The extracurricular committee completed several meetings to put the fundamental guidelines and regulations for planning, and designation of extracurriculars following the broad outlines of Kern’s steps [25] with some modifications named as modified Kern’s steps, these steps are described as follows:

Step 1: ***Problem identification*** through revising the report of extracurricular activities of the previous years and identifying the area of weakness and strength.

*Step 2:****Assessment of current needs****through* a) Studying the updated form of program mission, vision, and learning outcomes. b) Questionnaire to identify the students' interests. c) Methods such as informal discussions, focus group discussions, direct observation of skills, and strategic planning session.

*Step 3:****Select extracurricular activities****.* This is done through: a) Brainstorming of extracurricular academic ideas b) Analyzing the questionnaire to list the students' interests c) Determining which type of extracurricular activity is required to achieve the mission, vision, and program outcomes, d) Selecting other extracurriculars that fit the students' interests like sports, arts, and poems, e) Activation of the students' club, f) Availability of resources including funding, learning, and logistics materials. g) Alignment these ideas or activities with the modules and courses, h) Variation of extracurriculars according to year, phase, and level of the study considering the degree of difficulty of modules/courses and its requirements and assessments.

*Step 4:****Key performance indicators and target levels****:* Determination of the key performance indicators and target levels for the evaluation process is essential as it monitors and measures the level of achievement and reveals the impact of these activities on students ‘skills and all stakeholders included. The target levels should be revised ***periodically*** compared with internal and external benchmarks. KPIs can offer a regular way of monitoring proficiency in a variety of aspects of the learning and training procedure including extracurriculars [26,27].

*Step 5:****Implementation and monitoring of these activities****: this is done through the following:* a) Mapping of these activities through the longitudinal insertion of these activities in modules and courses of all phases and levels of the curriculum. Appropriate timing and hours are important. b) Distribution of the students into small groups with definite criteria for each c) Selection of faculty and staff members for guidance and observation, d) Participation of academic mentorship members. This will give more support and motivation to students to be more active participants. In addition, this will reflect more activation to career counseling, e) Invitation of eminent figures from the institute and community for some activities, f) Establishment of a rewarding system for active participants g) For good implementation, the extracurricular committee should: obtain permissions and facilities, identify, and allocate resources such as personnel: faculty, administrative/secretarial, logistic supports, and funding which may be direct funding or opportunity expenses. In addition to academic, stakeholder, and professional society support.

*Step 6:****Evaluation****:* This step is a crucial step in the quality cycle and provides information to the extracurricular committees to start the process of improvement and reform. Evaluation results can be employed to assess achievement, meet external requirements, and function as a nucleus for presentations and publications. The evaluation can be done through a) Questionnaire: this can be fulfilled by all ***stockholders*** (students, peers, employers, partnerships, and community members) involved in the activity through fulfilling a well-structured, valid, reliable questionnaire containing quantitative and qualitative sections b) Direct observation for all events and participants included in the activity.

**Fig. 1:**
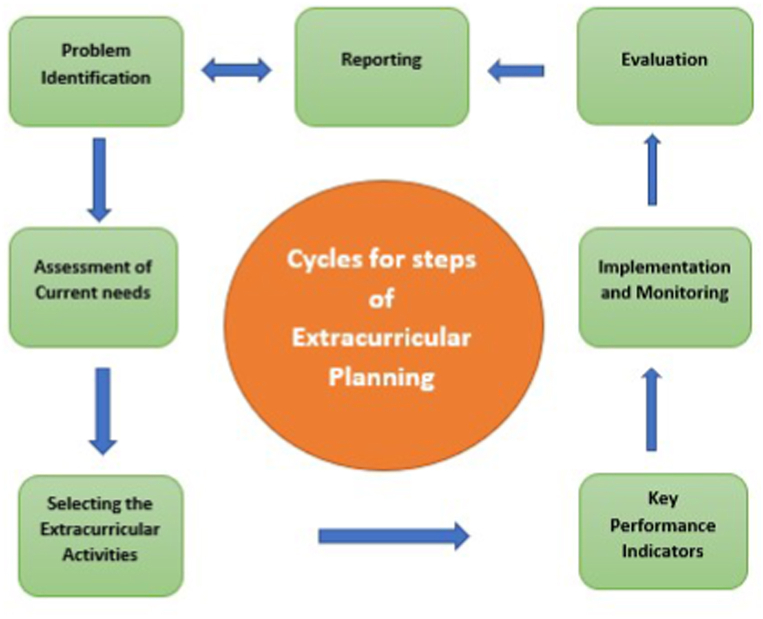
Steps of modified kern’s steps for planning the extracurricular activities.

Step 7: ***Reporting****:* A full detailed report for each activity should be done containing the number of active participants, beneficiaries, pros, cons, commendations, recommendations, and improvement action plan. These items were discussed in periodic meetings organized by the program hierarchy moderators and were documented and reported in the annual program report. These steps are represented in Fig. 1.

## 4 Application of the modified Kern' steps in the current study

### 4.1 Identification of the problem and assessment of the current situation and needs

The extracurricular committee holds several meetings to study the program mission, vision, and learning outcomes, aiming to identify the extent of running extracurricular activities in achieving goals, mission, vision, graduate attributes, and values at both institutional and program levels. Aiming to identify the current situation, need, and identification of gaps, the followings have occurred: a) As the Albaha Faculty of Medicine program is community-based, the extracurricular committee in collaboration with the medical education/vice deanship for quality and accreditation, and the students club made a questionnaire to investigate the students' opinions about the extent of their need for extracurriculars by making a list and choosing the most activities that help in achieving the objectives of the program. Therefore, the questionnaire was designed to have quantitative and qualitative parts. The validity and reliability of the questionnaire were confirmed by two cohort studies with two-week intervals in between. After cohort studies, the questionnaire was distributed electronically to all students. b) Several visits to the selected places such as healthcare providers, large malls, sports, social clubs, and government service providers were done aiming to select the optimal environment for the implementation of extracurricular activities to achieve goals and outcomes.

### 4.2 Formation of an extracurricular list with identified goals and KPIs

After identification of the students' needs, the extracurriculars were listed covering varieties of activities including volunteer and social activities, international and national days, research, sports, and others. The identified extracurriculars have well-defined goals and objectives which were fully described and aligned with the program outcomes. In addition, key performance indicators and target levels have been identified for each activity. Examples of these activities are listed in Table 2.

**Table 2:** List of extracurricular activities of volunteer and community services as offered by extracurricular committee member in association with students' club and program committee.

	Activities	Description and KPIs	Module	Alignment with CLOs	Alignment with PLOs
1.	The International Diabetes Day conference at the Al-Baha university main campus Alaqiq	The campaign included several awareness pillars for all segments of society in addition to the existence of a dedicated booth for estimation of blood sugar and dietetic management Time allocated: 1 day No. of student’s participants 24 No. of speaker representing our faculty 3 No. of audience 168 No. of attending faculty members: 13 No. of people who did estimation of blood sugar 100	Endocrine module/endocrinology and endocrine surgery module	2.2, 3.1,3.2,3.3	2.2,2.3,2.4,3.1,3.2
2.	Blood donation campaign in collaboration with the blood bank department King Fahad hospital	Awareness of the attendance about the importance of blood donation either in the donor himself or on the recipients No. of participants 110 No. of participating students 17 No. of faculty member speakers 2 No. of attending faculty members 9 No. of blood donors: 50	Blood module/hematology and oncology module	2.2,2.3,3.1,3.2,3.3	2.2,2.3,2.4,3.1,3.2
3.	International day for vision in collaboration with the ophthalmology department King Fahad hospital	The campaign included several awareness pillars for all segments of society in addition to the existence of a dedicated booth for estimation of visual disorders including early symptoms and signs, rapid diagnosis, and treatment Time allocated: 1 day No. of student’s participants 56 No. of speaker representing our faculty 3 No. of audience 200 No. of attending faculty members: 7 No. of people who did estimation of visual acuity and asked for advice: 110	Ophthalmology module	2.2,2.3,2.4,3.1,3.2,3.3	2.2,2.3,2.4,3.1,3.2
4.	Vaccination campaign in collaboration with the health affairs, Albaha province	The campaign included several awareness pillars for all segments of society in addition to the existence of a dedicated booth for importance of vaccination Time allocated: 1 day No. of student’s participants 19 No. of speaker representing our faculty 7 No. of audience: 170 No. of attending faculty members: 7 No. of people who received vaccination: 165	Primary health care module, Volunteer activities module, Child health module, Family medicine module	2,3,2.4,2.5,3.1,3.2,3.3	2.2,2.3,2.4,3.1,3.2
5.	The celebration of the National Saudi Day In faculty of medicine in both male and female sections	The Participation of the Faculty of Medicine in the celebration of the National Day and review some national achievements in all areas of development in accordance with the vision of the homeland 2030 Time allocated: 1 day No. of students participated 85 male and 60 females No. of audience 100; 95 In male and 59 in female section No. of attending faculty members: 51 male and 24 female	Volunteer activities, PHC,	3.1,3.2,3.3	2.2,2.3,2.4,3.1,3.2
6.	International Day of Colon cancer	The campaign included several awareness pillars for all segments of society in addition to the existence of a dedicated booth for awareness of risk factors, prevention, early diagnosis, and management Time allocated: 1 day No. of student’s participants 20 No. of speaker representing our faculty 2 No. of audience: 32 No. of attending faculty members: 7 No. of people asking examination and investigation: 23	Basic GIT module/Gastroenterology and GIT surgery module/Hematology and Oncology module	2.4,2.,3.1,3.2,3.3	2.2,2.3,2.4,3.1,3.2
7.	International Day of Breast cancer	The campaign included several awareness pillars for all segments of society in addition to the existence of a dedicated booth for awareness of risk factors, prevention, early diagnosis, and management of breast mass Time allocated: 1 day No. of student’s participants 23; 16 of them are female students No. of speaker representing our faculty 2 No. of audience: 32 No. of attending faculty members: 7 No. of females asking examination and investigation: 43	Reproductive module/family medicine/Hematology and Oncology module/women health module	2.3,2.4,3.1,3.2,3.3	2.2,2.3,2.4,3.1,3.2
8.	Child health campaign	Attendant students 37 male, 21 females No. of speaker representing our faculty 4 No. of audience: 64 No. of attending faculty members: 12 No. of families asking examination and investigation for their children: 35	Child health module	2.2,2.3,3.1, 3.2,3.3	2.2,2.3,2.4,3.1,3.2
9.	Smoking hazards	No. of student’s participants 41; 26 of them are female students No. of speaker representing our faculty 3 No. of audience: 42 No. of attending faculty members: 10 No. of persons asking examination and investigation:40	PHC, family medicine,	2.3,3.1,3.2	2.2,2.3,2.4,3.1,3.2
10.	Mental health campaign	No. of student’s participants 24; 18 of them are female students No. of speaker representing our faculty 2 No. of audience: 31 No. of attending faculty members: 11 No. of persons asking examination and investigation: 12	Mental health module	2.2,2.3,3.1, 3.2,3.3	2.2,2.3,2.4,3.1,3.2
11.	Support patients with renal diseases	No. of student’s participants 35; 26 of them are female students No. of speaker representing our faculty 2 No. of audience: 43 No. of attending faculty members: 5 No. of persons asking examination and investigation:49	Basic urinary system module/Nephrology and urology module	2.2,2.3,3.1, 3.2,3.3	2.2,2.3,2.4,3.1,3.2
12.	Down syndrome	No. of student’s participants 21; 15 of them are female students No. of speaker representing our faculty 2 No. of audience: 37 No. of attending faculty members: 10 No. of families asking examination and investigation:7	Child health module, Nutrition module	2.3,3.1,3.2,3.3	2.2,2.3,2.4,3.1,3.2
13.	Osteoporosis	No. of student’s participants 23; 16 of them are female students No. of speaker representing our faculty 2 No. of audience: 43 No. of attending faculty members: 17 No. of females asking examination and investigation:49	Family medicine module, women health module	2.3,3.1,3.2,3.3	2.2,2.3,2.4,3.1,3.2

### 4.3 Implementation phase

All extracurriculars were mapped in the schedule and aligned with modules. The required permissions, facilities, resources, and funding for each activity have been discussed, identified, and allocated.

### 4.4 Evaluation

The extracurriculars were evaluated using another Likert scale questionnaire to evaluate the degree of students' and personnel' satisfaction.

## 5 Results

### 5.1 Initial assessment of current situation and needs using initial questionnaire

Analysis of the current situation using the initial questionnaire revealed that about 71 out of 184 (38.58%) showed satisfaction about the current extracurriculars, of these 42 out of 116 (36.1%), 29 out of 68 (42.6%) for male and female sections, respectively. Further details are discussed in Table 1.

Analysis of the qualitative part of the questionnaire reported the followings: lack of interest by some students and personnel; not aligned with some courses and modules, not suitable for the phase and level of the curriculum, the time specified for each activity did not correspond to the nature of activity and the desired objectives, the timing is not suitable as it is close to the time of assessments, interfere with student-centered activities such as problem-based and self-directed learning, costive for some students, preparation for some activities was much exhaustive, lack of cooperation with peers, and lack of orientation, guidance, and support.

### 5.2 Results of the questionnaire applied for re-evaluation stage

This phase was evaluated by the second questionnaire which was delivered electronically to 447 students representing all academic years for both male and female sections. Four hundred and four students responded to the questionnaire. The distribution of the current study of students was as follows: phase I (93, 23%) included 59 males, and 34 females, phase II (151, 37.4%) included 86 males and 56 females, and phase III (160, 39.6%) included 87 male and 73 female. In addition, these students were further subdivided according to their achievement into high 140 (34.7%), moderate 134 (33.2%), and low grade 130 (32.2%).

About 270 out of 404 (66.8%) were satisfied. Of these 159 out of 232 (68.4%), and 111 out of 172 (64.5%) for male and female, respectively with a significant difference p-value 0.0036. Regarding satisfaction and students' achievement, 95 out of 140 (67.8%) was of high-grade achievers, 88 out of 134 (65.7%) for moderate, and 87 out of 130 (66.9%) for low-grade achievers. By comparison of the students in the three phases, a significant P = 0.00 was obtained. These figures are further discussed regarding the male and female sections in Table 3, Table 4.

**Table 3:** Distribution of the students according to gender, phase of the program and students' achievement in the evaluation stage.

Variables		Gender; No. and percentage				Total	*p-value (Student t-test)*	*t-value (Student t-test)*
Gender		Male		Female				
		No	%∗	No	%∗			
		232	57.4	172	42.6	404		
Phase	I	59	25.4	34	19.8	93	0.00	0.49
	II	86	37	65	37.8	151		
	III	87	37.5	73	42.4	160		
Grade	High	81	34.9	59	34.3	140	0.0	0.5
	Moderate	73	31.5	61	35.5	134		
	Low	78	33.6	52	30.2	130		

∗ Percentage of number of students to its actual number in both male and female section.

**Table 4:** Distribution of the degree of satisfaction concerning gender and program phase in the evaluation stage.

Phase of the program	Gender	Total	Strongly satisfied		Satisfied		Dissatisfied		Strongly Dissatisfied		P value (*t*-test)
			No.	%	No.	%	No.	%	No.	%	
	Male	232	93	40	66	28.4	36	15.5	37	15.9	0.18
	Female	172	59	34.3	52	30.2	28	16.3	33	19.2	
I	Male	59	24	40.6	17	28.8	10	16.9	8	13.6	0.30
	Female	34	13	38.2	11	32.4	3	8.8	7	20.9	
II	Male	86	36	41.8	25	29.1	14	16.3	11	12.8	0.13
	Female	65	22	33.8	21	32.3	11	16.9	11	16.9	
III	Male	87	33	37.9	24	27.9	12	13.8	18	20.9	0.26
	Female	73	24	32.8	20	27.4	14	19.2	15	20.5	

There are no significant associations between students' satisfaction with extracurricular activities between males and females within the phases of the program. All data and P values are described in Table 4.

Furthermore, the students in phase I showed the greatest satisfaction among phases with significant difference (P = 0.00). The highest student satisfaction was found in male students representing phase II (70.9%), followed by female students in phase I (70.6%). The lowest student satisfaction was seen in the female section of phase III. The details are mentioned in Table 4, Table 5.

**Table 5:** Summary of the students' satisfaction according to program phases in the evaluation stage.

Section/phase	No. and % of satisfaction		No. and % of Dissatisfaction		p-value (Student t-test)	t-value (Student t-test)
Male section	159	68.5	73	31.5	**0.00**	**11.66**
Female section	111	64.5	61	35.5		
Phase I	65	69.9	28	30.1	**0.00**	**11.54**
Phase II	104	68.9	47	31.1		
Phase III	101	63.1	59	36.9		
Male students of phase I	41	69.5	18	30.5	**0.00**	**14.68**
Male students of phase II	61	70.9	25	29.1		
Male students of phase III	57	65.5	30	34.5		
Female students of phase I	24	70.6	10	29.4		
Female students of phase II	43	66.2	22	33.8		
Female students of phase III	44	60.3	29	39.7		

Using one-way ANOVA test for comparing the results of all phases including male and female sections on students' grades for both sections. A significant p-value was obtained, P = 0.00.

Further analysis for both male and female sections:

***Grade and students' satisfaction:* in male section** using *t*-test for the students ‘grades with the degree of satisfaction, we found significance with a P = 0.00. Also, in ***female section***, a significant P = 0.00.

**Phase and students’ satisfaction:** Comparison between males and females regarding grades and students' satisfaction; a significant difference was obtained in phase I (P = 0.02), while no significances were obtained in either phase II (P = 0.43) or phase III (P = 0.11). ***In the male section:*** no significant differences were obtained between students' grades and students' satisfaction in phases I, II, and III with a P = 0.4, P = 0.98, and P = 0.096, respectively. ***Phases and students' grades in the female section:*** no significant differences were obtained between students' grades and degree of satisfaction in phases I, II, and III with P = 0.61, 0.82, and 0.84, respectively.

***Phases and students' grade between male and female sections using t-test revealed that*** in phase I, a significant difference was obtained in students' satisfaction between male and female in low-grade students of phase I and phase III with a P = 0.02, 0.02, respectively. Otherwise, no significance was obtained.

***Comparison of student's grade with that counterpart in all phases with students' satisfaction using ANOVA:*** (1) **high grade** in the male section**;** there is a significant difference with P = 0.04, while in the female section, no significance was obtained P = 0.25. (2) **moderate grade,** no significance was obtained either in male P = 0.18 or in female section P = 0.22. (3) **low grade,** no significant was obtained in both male and female sections P = 0.91, and 0.23, respectively.

### 5.3 Comparison between the results of both questionnaires

**Fig. 2:**
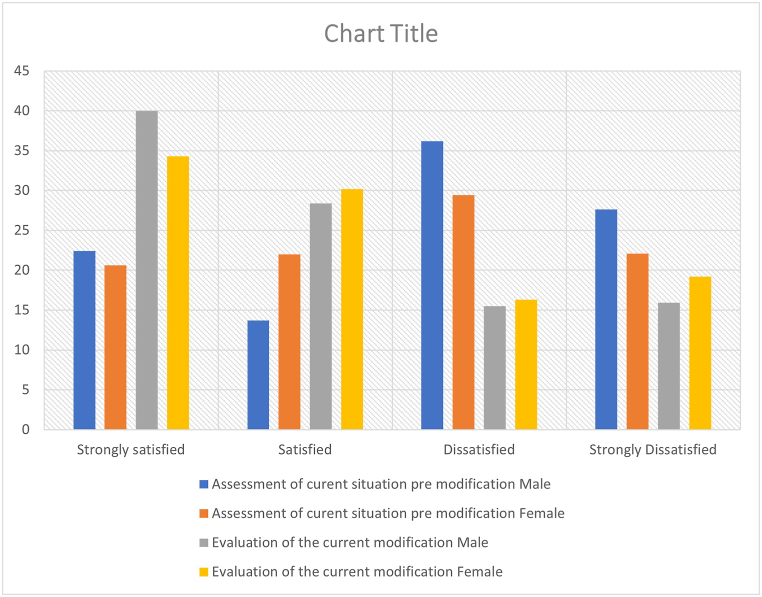
Students' satisfaction between pre and post modification in male and female section.

**Fig. 3:**
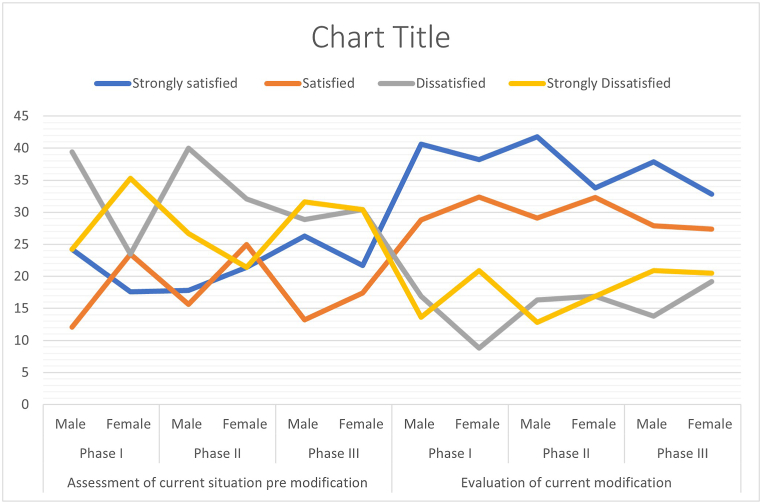
Students' satisfaction regarding phases of the program in pre and post modification for male and female sections.

Comparison between students' satisfaction that resulted in the initial questionnaire with that appeared in the questionnaire of the evaluation stage revealed a significant difference with a P = 0.00 (Fig. 2, Fig. 3).

In all tables, due to the asymmetrical representation of both male and female sections, the number and percentage were related to the total number of each section. In addition, the percentage of satisfaction was calculated relative to the number of students in each section. Furthermore, concerning the phase of the program, the percentage described in each raw was closely related to the number of students for each phase either in both male and female sections.

## 6 Discussion

This study showed that students' satisfaction with extracurricular activities was weak, while it increased significantly after the implementation of the improvement plan, to about 60%. This satisfaction is not only among students with high levels of achievement but among all levels. This means that this improvement plan was reflected not only in the students' satisfaction but also in the environment of the extracurriculars within the program.

In the current research, some students registered their objection to the time of extracurricular activities in the timetable, as well as the time specified for each activity, which may not be compatible with the nature of the activity. This observation is also found in the study of Whiteley & Richard [28] who found that a significant majority of teachers do not supervise extracurricular activities when they have no preparation time and recommended the importance of timing of extracurriculars in achieving their goals.

The time allocated for extracurriculars should be adjusted carefully. The direct relationship between hours of extracurriculars and student well-being has been recognized [29,30]. This may reflect a negative effect on academic performance if the students expended more hours participating in these activities, especially those of social interest that are time and energy-consuming [1]. Furthermore, an imbalance in academic requirements and extracurriculars may lead to depressive symptoms that in turn reflect on low academic achievement [31,32]. In this instance, good time management is recommended to decrease the low academic achievement and stress among the participants [[33], [34], [35], [36]].

Participation in extracurricular activities supports the development of appropriate nonacademic talents that promote improving clinical skills and student performance [37]. Another study revealed that extracurricular participants have better skills in terms of reflection, communication, collaboration, decision-making, leadership, responsibility, and commitment and these skills appear early in the academic year [20,38] and become more prominent in clinical years [39,40].

In the present study, we found that the sharing of students in extracurricular activities is increased in the clinical years than in basic years, reaching the highest in the 5th academic year. This contrasts with other studies which found positive associations between extracurriculars and stress among second [41,42], and third year [42,43] students owing to increased extracurricular activities and academic overload. This is due to the nature of the academic curriculum and the alignment of these activities with the modules in these years. So, educators should take into account the potential impact of preserving extracurricular activities on students' health when designing and planning academic courses [44].

The reform plan that was conducted in our program was three-dimensional that included extracurricular activities, learning and teaching, and assessment methods. The plan was run in parallel in all dimensions and specific key performance indicators {KPIs) with internal and external benchmarks applied. The reform committee conducted several meetings, interviews with the students, faculty, academic staff, and stakeholders, and reviews of annual course reports, as well as reports of the extracurriculars, and psychometric analysis of all assessment methods. The obtained results were desirable, encouraging, and motivating, in addition to the high satisfaction of the students towards the comprehensive reform plan that was adopted.

## 7 Conclusion

The rate of student satisfaction with the activities has improved significantly after the implementation of the improvement plan, and satisfaction was high among all students, regardless of their academic achievement. This will inevitably lead to the achievement of the objectives of these activities, which will reflect positively on the improvement of the student's academic performance.

Extracurricular activities might be flexible and undergo periodic changes related to the nature of the modules and courses of the curriculum. Insertion of these activities within the modules must be matched with the learning outcomes and might consider the degree of difficulty of running modules and assessments.

Following the cycle of developing extracurricular activities in designing, implementing, monitoring, evaluating, and reporting, the extracurricular activities will be more efficient in enhancing of learning climate and make the process of learning more enjoyable, especially in a solid medical integrated curriculum. In addition, time management of these activities is essential to avoid adverse effects such as low academic activity and low student performance.

## 8 Limitations of the study

The different number of respondents for the initial and re-evaluation stages may be explained by the appearance of a strong incentive for students to participate in the second questionnaire, after implementing the reform plan, and this is considered an indirect success for the reform plan, and this incentive led to an increase in the number of students participating in the second questionnaire, which created a difference between the number of students in the first and second questionnaires. Accordingly, the percentage was used, not the number, in the comparisons made in this study. So, this difference in participants is considered as a limitation of the study.

## Author contribution statement

Ihab Shafek Atta: designed the study, analyzed the results, and interpreted the data and revising the final manuscript.

Ali Hendi Alghamdi: distribution of questionnaire, monitoring the process, collecting the data and revising the final manuscript.

Rajab A. Alzahrani: revising the data, perform the statistical analysis, wrote the draft and revising the final manuscript.

## Funding statement

This research did not receive any specific grant from funding agencies in the public, commercial, or not-for-profit sectors.

## Data availability statement

The authors do not have permission to share data.

## Declaration of interest’s statement

The authors declare no conflict of interest.

## Ethical approval

This research was implemented after taking the ethical approval from the assigned committee of Albaha ethical committee under reference number 62/2020. The informed consent was taken from all participants included in the study.
