# Supplementary material for: Optimal Steps for designing and implementing the extracurriculars through the integrative medical approach
Source: Heliyon. 2023 Feb 15;9(3):e13755. doi: 10.1016/j.heliyon.2023.e13755 (PMC9988501; doi:10.1016/j.heliyon.2023.e13755)
Supplement: Multimedia component 1 [file mmc1.docx]

Questionnaire measure the students’ satisfaction about the extracurriculars in MBBS program of Albaha faculty of medicine.

|  | Item | Strongly satisfied  (5) | Satisfied  (4) | Neutral  (3) | Dissatisfied  (2) | Strongly dissatisfied  (1) |
| --- | --- | --- | --- | --- | --- | --- |
|  | The extracurriculars reflect the vision, mission, and goals of the faculty and institution |  |  |  |  |  |
|  | The extracurriculars are in alignment with courses and modules of the curriculum. |  |  |  |  |  |
|  | The extracurriculars are matched with the phases and levels of the MBBS program |  |  |  |  |  |
|  | The extracurriculars have well defined objectives and outcomes. |  |  |  |  |  |
|  | The extracurriculars are suitable for the nature of the curriculum. |  |  |  |  |  |
|  | The extracurricular have well defined allocated hours in the MBBS program. |  |  |  |  |  |
|  | The extracurriculars help the students in achieving the outcome of the MBBs program |  |  |  |  |  |
|  | The extracurriculars promote self-learning which is one of criteria of the MBBS program |  |  |  |  |  |
|  | The faculty encourage the students to share in the extracurriculars. |  |  |  |  |  |
|  | The list of extracurriculars contains wide varieties of activities as sports, research, scientific meetings, and workshops. |  |  |  |  |  |
|  | The time spend in the extracurricular is suitable for practicing these activities. |  |  |  |  |  |
|  | The extracurriculars help in the development of knowledge among the students. |  |  |  |  |  |
|  | The extracurriculars motivate the acquisition of communication, and interpersonal skills. |  |  |  |  |  |
|  | The extracurriculars alleviate the psychomotor skills among students through practicing, and monitoring, and direct feedback. |  |  |  |  |  |
|  | The extracurriculars enhance the communication among students, faculty, and administrative members. |  |  |  |  |  |
|  | The extracurriculars contains activities that help the low achievers’ students and enhance their performance. |  |  |  |  |  |
|  | The funding and logistic materials applied for the extracurriculars are well allocated and easily obtained. |  |  |  |  |  |
|  | The faculty honors the distinguished students in the extracurriculars. |  |  |  |  |  |
